# MiR‐335‐5p inhibits the progression of head and neck squamous cell carcinoma by targeting MAP3K2

**DOI:** 10.1002/2211-5463.12955

**Published:** 2020-10-12

**Authors:** Zhenxiao Wang, Shuoqing Yuan, Xiaoming Cao, Chaoping Huang, Aobo Zhang, Cheng Lu, Liangfa Liu

**Affiliations:** ^1^ Department of Otolaryngology Head and Neck Surgery Beijing Friendship Hospital Capital Medical University Beijing China; ^2^ Department of Otolaryngology Dezhou People‘s Hospital Dezhou China

**Keywords:** antitumor, head and neck squamous cell carcinoma, MAP3K2, miR‐335‐5p

## Abstract

Mounting evidence has indicated that aberrantly expressed microRNAs (miRNAs) play key roles in tumorigenesis, including in head and neck squamous cell carcinoma (HNSCC). Previous studies have shown that miR‐335‐5p can serve as a tumor suppressor or an oncogene in cancer. However, the clinical importance and biological effects of miR‐335‐5p in HNSCC have not been determined. Here, we investigated the expression pattern, functional role, and mechanisms of miR‐335‐5p in HNSCC. We showed a decreased expression of miR‐335‐5p in HNSCC samples from the TCGA and GEO databases. Consistently, we detected a downregulation of miR‐335‐5p in HNSCC cell lines and patient tissues. The expression of miR‐335‐5p was inversely correlated with advanced clinical TNM stage and lymph node metastasis in HNSCC patients. miR‐335‐5p overexpression inhibited HNSCC cell proliferation and induced apoptosis, while miR‐335‐5p inhibition had the opposite effects. miR‐335‐5p overexpression suppressed tumor growth in mice. Bioinformatic analyses and functional assays identified *MAP3K2* as a target of miR‐335‐5p, and we showed that miR‐335‐5p downregulated mitogen‐activated protein kinase kinase kinase 2 (MAP3K2) expression in HNSCC cells. We found an inverse association between *MAP3K2* and miR‐335‐5p expression in 38 pairs of HNSCC tissues. Furthermore, the effect of miR‐335‐5p overexpression on growth and metastasis as well as cell apoptosis in HNSCC cells could be partially rescued by MAP3K2 expression. Collectively, our data show that miR‐335‐5p inhibits the development of HNSCC by regulating MAP3K2 expression. Thus, these findings offer novel insights into a potential therapeutic strategy for HNSCC patients.

## Graphical abstract

This study demonstrated that miR‐335‐5p expression was reduced in HNSCC. miR‐335‐5p expression could inhibit HNSCC cell growth, metastasis, and promote cell apoptosis while it could suppress tumor growth *in* *vivo*. We identified *MAP3K2* as a novel target of miR‐335‐5p and MAP3K2 overexpression partially rescued the inhibitory role of miR‐335‐5p. Hence, miR‐335‐5p might constitute a potential therapeutic target for HNSCC patients.

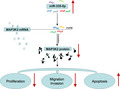

**Abbreviations**
CCK8: cell counting kit‐8
EdU: 5‐ethynyl‐2′‐deoxyuridine
EMT: epithelial–mesenchymal transition
HNSCC: head and neck squamous cell carcinoma
MAP3K2: mitogen‐activated protein kinase kinase kinase 2
miRNA: microRNA
NC: negative control
qRT–PCR: quantitative real‐time PCR

Head and neck squamous cell carcinoma is a common malignancy worldwide and is characterized by high morbidity and mortality [1]. HNSCC consists of different anatomical structures with distinct biological characteristics [2]. Nearly half a million new cases of HNSCC are diagnosed annually worldwide [3], and most cases are middle‐aged and elderly males [4]. Research has indicated that tobacco, alcohol, and human papillomavirus infection are hazard factors for HNSCC [5, 6]. Unfortunately, despite the rapid development of surgical treatments and radiochemotherapy in the past years, the 5‐year survival rate for HNSCC remains unsatisfactory [7]. Tumor metastasis and relapse are the major causes of treatment failure for HNSCC [8], and ~ 70% of patients show recurrent or metastatic disease [8]. Furthermore, more than half of patients are at advanced stage at diagnosis, which likely contributes to the high cancer‐related fatality rate [9]. Hence, it is critical to investigate the molecular mechanisms that regulate HNSCC metastasis and recurrence, as better understanding of these mechanisms could provide novel therapeutic strategies for HNSCC.

MicroRNAs (miRNAs), a type of noncoding RNA, consist of 18–25 nucleotides and modulate gene expression by inhibiting translation or mRNA degradation through binding the 3′UTR of target genes [10]. Previous studies have demonstrated that some miRNAs are aberrantly expressed in cancer and participate in tumor initiation and development with roles in cell proliferation, metastasis, or apoptosis [11, 12]. Moreover, miRNAs can serve as either oncogenes or tumor suppressors in cancers. For instance, the miR‐200b/LAMA4 pathway suppresses tumor metastasis in renal cell carcinoma [11] and the miR‐9/HMGA2 axis restrains carcinogenesis in hepatocellular carcinoma [12], but the miR‐92a/KLF4 signaling facilitates cell growth and invasion in glioma [13]. Mounting evidence has demonstrated abnormal miR‐335‐5p expression and function in multiple cancer types; ectopic miR‐335‐5p can significantly promote or inhibit the malignant characteristics of cancer cells, depending on the cancer type, indicating that miR‐335‐5p might play a vital part in tumor development [14, 15, 16, 17, 18]. However, the biological function and mechanisms of miR‐335‐5p in HNSCC remain largely unknown.

In this study, we investigated the clinical significance, biological role, and molecular mechanisms of miR‐335‐5p in HNSCC. Our data indicated that miR‐335‐5p expression was conversely related to advanced TNM stage and lymph node metastasis in HNSCC patients. We also found that miR‐335‐5p suppressed growth and metastasis and induced apoptosis of HNSCC cells by targeting mitogen‐activated protein kinase kinase kinase 2 (MAP3K2). These findings provide novel and potentially beneficial insights into treatment strategies for this disease.

## Materials and methods

### Patients and specimens

Thirty‐eight pairs of human HNSCC tissues and paired normal tissues were obtained from Beijing Friendship Hospital from 2017 to 2019. All patients signed informed consent, and no patient received radiochemotherapy before operation. All specimens were immediately stored in liquid nitrogen for further use. This study was approved by the Ethics Committee of Beijing Friendship Hospital, Capital Medical University, and conformed to the standards set by the Declaration of Helsinki.

### Cell culture

Human HNSCC FaDu, CAL‐27 and TU212 cell lines, and the bronchial mucosa BEAS‐2B cell line were obtained from the Cell Bank of Chinese Academy of Sciences (Shanghai, China). Cells were cultured in RPMI‐1640 (Sigma‐Aldrich, St. Louis, MO, USA) supplemented with 10% FBS and 1% antibiotics at 37 °C in a humidified chamber with 5% CO_2_.

### Reagents and cell transfection

miR‐335‐5p mimic, inhibitor, the MAP3K2 overexpression vector pcDNA3.1‐MAP3K2, and negative controls (NC) were designed by RiboBio (Guangzhou, China). Oligonucleotides were transfected into cells using Lipofectamine 2000 (Invitrogen, Carlsbad, CA, USA), following the manufacturer's instructions. The miR‐335‐5p overexpression vector was designed and embedded into lentiviruses by Hanbio (Shanghai, China). The presence of Polybrene is needed for lentiviruses transfection.

### Quantitative real‐time PCR (qRT–PCR)

Total RNA was extracted from HNSCC cells using TRIzol Reagent (Sigma) following the manufacturer's instructions. For evaluation of miR‐335‐5p, reverse transcription and quantification were conducted using the Bulge‐Loop miRNA qRT**–**PCR Starter Kit (RiboBio). For evaluation of *MAP3K2* mRNA, reverse transcription was performed by the Reverse Transcription Kit (Roche, Switzerland). PCR was performed using the SYBR Green Master Kit (Roche, Rotkreuz, Switzerland). *U6* or *GAPDH* mRNA served as internal controls. Gene expression was calculated using the $2^{‐\Delta \Delta C_{t}}$ method. The primer sequences are listed in Table 1.

**Table 1:** Primers used for quantitative real‐time RT–PCR analysis. F, forward; R, reverse.

Gene	Primer sequence 5′–3′
Hsa_miR‐335‐5p	F: TGTTTTGAGCGGGGGTCAAG R: TGAATATAGCAAATGAGAGG
U‐6	F: CTCGCTTCGGCAGCACATATACT R: CGGCTGCAGATGAGATAG
MAP3K2	F: CCCCAGGTTACATTCCAGATGA R: GCATTCGTGATTTTGGATAGCTC
GAPDH	F: GAGTCAACGGATTTGGTCGT R: GACAAGCTTCCCGTTCTCAG

### Western blotting

Cells were washed three times and lysed in lysis buffer, and the protein concentrations were determined using the BCA Assay Kit (Beyotime, Jiangsu, China). Protein samples were separated by 10% SDS/PAGE and then transferred to poly(vinylidene difluoride) (PVDF) membranes (Bio‐Rad, Hercules, CA, USA). The PVDF membranes were incubated with human MAP3K2 (1 : 1000 dilution; Cell Signaling Technology, Boston, MA, USA), human P53 (1 : 1000 dilution; Cell Signaling Technology), N‐cadherin (1 : 1000 dilution; Cell Signaling Technology), E‐cadherin (1 : 1000 dilution; Cell Signaling Technology), and GAPDH (1 : 2000 dilution; ZSGB‐BIO, Beijing, China) at 4 °C overnight, followed by incubation with secondary antibodies for 100 min. Enhanced chemiluminescence reagent (Tanon, Shanghai, China) was used to visualize protein bands.

### Cell Counting Kit‐8 (CCK‐8) assay

Cell growth was detected by cell counting kit‐8 (CCK8) assay. Transfected cells (2000 cells/well) were cultured in 96‐well plates. Cells were incubated for 24, 48, and 72 h. Cells were then incubated with 10% CCK8 solution (Dojindo, Tokyo, Japan) and incubated for 60 min. Absorbance was detected at 450 nm using an ELISA plate reader (Bio‐Rad).

### Colony formation assay

After transfection, cells were plated in 6‐well plates (1000 cells/well) and cultured in a 37 °C incubator. Ten days later, the plates were washed and fixed with 4% paraformaldehyde, and cells were stained with 0.1% crystal violet. The numbers of colonies were then counted.

### 5‐Ethynyl‐2′‐deoxyuridine (EdU) assay

Cell growth was also measured with the EdU Apollo643 Kit (RiboBio) following the manufacturer's protocol. After transfection for 48 h, the cells were seeded into 24‐well plates (1 × 10^5^ cells/well). Cells were cultured with 50 μm EdU for 2 h, fixed with 4% paraformaldehyde for 30 min, and then dyed by Apollo643 for 30 min. Finally, cells were dyed by Hoechst 33342. Cells were then examined by confocal fluorescence microscopy.

### Wound‐healing scratch assay

Cells were cultured to 80–90% confluence in 6‐well plates. A standard 10‐μL pipette tip was used to make a scratch in the cell monolayer. The plates were washed twice to remove any floating cells. Fresh medium with low concentration of FBS was then added. After 36 h, the wound areas were examined. Images of the wound‐healing process were captured for analysis.

### Transwell assays

Transwell assays were performed to evaluate the migration or invasion abilities of transfected cells. For invasion assays, Matrigel was added to upper chambers. Cells (2 × 10^5^ cells/well) were plated into the chambers, and 750 μL RPMI‐1640 with 20% FBS was added to the lower chambers. After 24 h, the nonmigrated or noninvaded cells were removed with cotton swabs. The adherent cells were fixed in 4% paraformaldehyde and dyed by crystal violet. The migrated or invaded cells were observed by microscopy and counted.

### Dual‐luciferase reporter assay

The wild‐type 3′UTR fragment of *MAP3K2* containing the putative miR‐335‐5p binding sites was subcloned into the luciferase vector pSI‐Check2 (Promega, Madison, WI, USA) to construct pSI‐Check2‐WT. The mutant 3′UTR fragment of *MAP3K2* with mutations in the putative binding sites was synthesized and subcloned to construct pSI‐Check2‐Mut. The vectors were confirmed by sequencing. We cultured 293T cells to 50%–70% confluence in 96‐well plates. Next, 5 pmol miR‐335‐5p mimic or NC and 0.16 µg pSI‐Check2‐WT or pSI‐Check2‐Mut vector was cotransfected into 293T cells using Lipofectamine 2000 (Invitrogen). After 24 h, the cells were lysed with passive lysis buffer; 20 μL cell lysate was mixed with 100 μL Luciferase Assay Reagent II (Promega), and firefly luciferase activity was measured. Renilla luciferase intensity was detected as an internal control.

### Apoptosis analysis

Transfected cells were digested and stained with 2 µL PE and 2 µL 7‐AAD (BioLegend, San Diego, CA, USA) in a darkroom at room temperature for 15 min. Data were obtained by flow cytometer and analyzed by flowjo software (BD Biosciences, San Jose, CA, USA).

### Animal experiments

BALB/c nude mice (5 weeks old) were purchased from Beijing Vital River Laboratory Animal Technology Co., Ltd (Beijing, China). FaDu cells (100 µL, 5 × 10^7^ cells/mL) transfected with miR‐335‐5p overexpression‐lentivirus or NC‐lentivirus were injected in the right flanks of each mouse (*n* = 5 in each group). Tumor growth was observed every 7 days until 1 month after injection, and the volume of tumors was measured by the formula: *V* = length × width^2^ × 0.5. Animal protocols were supervised by the Institutional Animal Care and Ethics Committee of the Beijing Friendship Hospital, Capital Medical University.

### Statistical analysis

Data are shown as the mean ± SD of triple independent experiments. graphpad prism 8 (GraphPad Software, San Diego, CA, USA) or spss 22.0 software (IBM, Chicago, IL, USA) was used to analyze the data. Statistical analysis was calculated with Student's *t*‐test between two groups; one‐way ANOVA was used to detect the differences among three or more groups. *P* < 0.05 was considered statistically significant.

## Results

### miR‐335‐5p expression is decreased in HNSCC

**Fig. 1:**
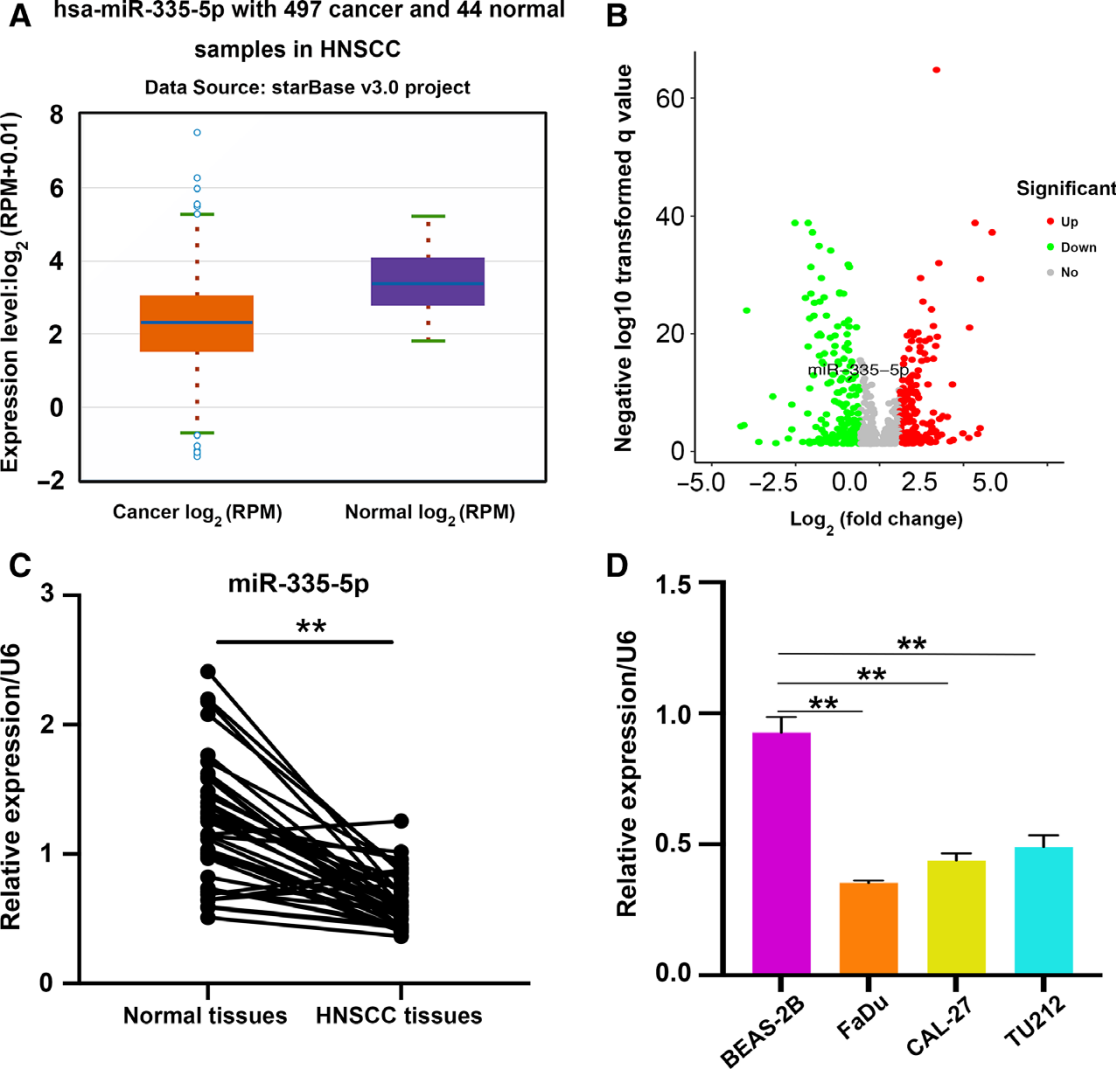
miR‐335‐5p is downregulated in HNSCC. (A) qRT–PCR was used to examine the expression levels of miR‐335‐5p in 497 HNSCC tissues and 44 normal tissues with TCGA and GEO databases. (B) Differential miRNA expression between 518 HNSCC tissues and 44 normal tissues. The volcano plot shows upregulated (red points) and downregulated miRNAs (cyan points), screened on the basis of log_2_(fold change) > 1.0 and a correction for *P* < 0.05. The gray points represent miRNAs with no significant difference. (C) miR‐335‐5p was examined in 38 paired of HNSCC tissues and adjacent normal tissues by qRT–PCR. (D) miR‐335‐5p expression in HNSCC cell lines and BEAS‐2B cells was analyzed by qRT–PCR (*n = 3*). Data are presented as the mean ± SD. Comparisons between groups were analyzed using Student's *t*‐test. **P < 0.01.

To first explore whether miR‐335‐5p is expressed in HNSCC, we investigated the expression patterns of miR‐335‐5p from the TCGA Database with StarBase V3.0 [19] and the combination of GEO and TCGA Database with dbDEMC V2.0 (http://www.picb.ac.cn/dbDEMC/). miR‐335‐5p expression was downregulated in HNSCC tissues compared with the normal controls (Fig. 1A,B). We also detected the miR‐335‐5p expression in 38 pairs of HNSCC samples from our hospital and found that the expression levels of miR‐335‐5p were decreased in HNSCC tissues compared with controls (Fig. 1C), which was consistent with the findings in the TCGA database. Similar results were observed in HNSCC cell lines, comparing to BEAS‐2B cells (Fig. 1D). We next analyzed the association between miR‐335‐5p expression and clinicopathological characteristics of HNSCC patients. The low expression of miR‐335‐5p correlated with clinical TNM stage and lymph node metastasis in HNSCC patients (Table 2

). Together, our results demonstrated that miR‐335‐5p expression is reduced in HNSCC and indicate that miR‐335‐5p may repress the development of HNSCC.

**Table 2:** Association between the miR‐335‐5p expression and clinicopathological characteristics of HNSCC. Low/high by the sample median. Pearson’s chi‐square test. *P* < 0.05 was considered statistically significant.

Characteristics	Cases	miR‐335‐5p		*P* value
	(*n* = 38)	Low (*n* = 25)	High (*n* = 13)	
Age (years)				
＜ 60	17	11	6	0.367
≥ 60	21	14	7	
Smoking				
No	5	2	3	0.296
Yes	33	23	10	
Lymph nodes metastasis				
No	12	3	9	0.004*
Yes	26	22	4	
Tumor differentiation				
Well‐moderate	25	16	9	0.459
Poor	13	9	4	
TNM stage				
Ⅰ—Ⅱ	15	7	8	0.011*
Ⅲ—Ⅳ	23	18	5	

^*^ *P*＜0.05.

## miR‐335‐5p inhibits HNSCC cell growth and induces apoptosis

**Fig. 2:**
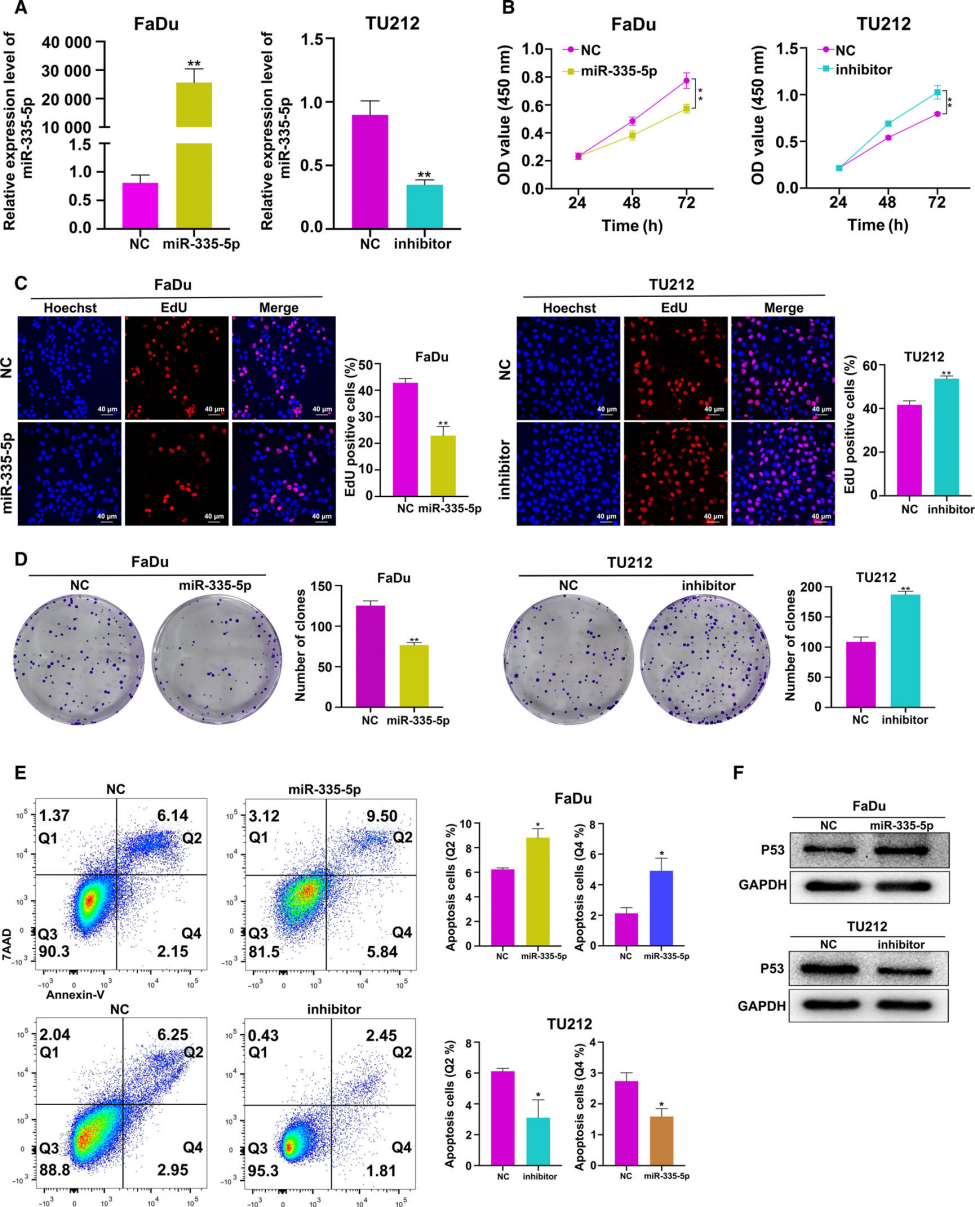
miR‐335‐5p inhibits HNSCC cell growth and induces apoptosis. (A) Transfection efficiency of the miR‐335‐5p mimic and inhibitor in HNSCC cells (*n = 3*). (B, C) Effects of miR‐335‐5p mimic and inhibitor on HNSCC cell proliferation as determined by CCK8 (*n = 3*) and EdU assays (*n = 3*; scale bar: 40 μm). (D) Representative colony formation assay showing clonogenic survival in HNSCC cells transfected as indicated (*n = 3*). (E) Flow cytometry was used to explore the effects of miR‐335‐5p on cell apoptosis. Q2 represented the late apoptosis of HNSCC cells, Q4 represented the early apoptosis of HNSCC cells (*n = 3*). (F) Western blot assay was used to detect the expression of p53 protein in HNSCC cells with miR‐335‐5p overexpression and downregulation (*n = 3*). Data are presented as mean ± SD. Comparisons between groups were analyzed using Student's *t*‐test. *P < 0.05, **P < 0.01.

To further explore the functional role of miR‐335‐5p in HNSCC, miR‐335‐5p mimic, inhibitor or scramble controls were transfected in HNSCC cells. Because of the low expression of miR‐335‐5p in FaDu cells and the relatively high expression of miR‐335‐5p in TU212 cells, FaDu and TU212 cells were used in these experiments. qRT**–**PCR confirmed the effectiveness of miR‐335‐5p mimic and inhibitor in FaDu and TU212 cells (Fig. 2A). We evaluated cell proliferation using CCK8 assay and found that miR‐335‐5p overexpression suppressed cell growth in FaDu cells, while miR‐335‐5p inhibitor in TU212 cells resulted in increased cell growth (Fig. 2B). EdU assay also confirmed that FaDu cell proliferation was reduced by miR‐335‐5p overexpression as indicated by the ratio of EdU‐positive cells, while transfection of TU212 cells with the miR‐335‐5p inhibitor led to increased proliferation (Fig. 2C). Similarly, colony formation ability of FaDu cells was suppressed by miR‐335‐5p overexpression, and vice versa in TU212 cells (Fig. 2D).

To determine whether miR‐335‐5p‐mediated reduction in cell growth involved apoptosis induction, we evaluated apoptosis by flow cytometry. The apoptotic ratios of Q2 and Q4 in miR‐335‐5p overexpression groups were higher than that of normal controls, but the apoptotic ratios of Q2 and Q4 in miR‐335‐5p decrease groups were lower than that of normal controls. The results demonstrated that miR‐335‐5p overexpression elevated the ratio of apoptotic cells in FaDu, while the miR‐335‐5p inhibitor reduced the population of apoptotic cells in TU212 (Fig. 2E). Moreover, the apoptosis‐related protein p53 was increased in FaDu cells overexpressing miR‐335‐5p, but was downregulated in TU212 cells after transfection with the miR‐335‐5p inhibitor (Fig. 2F). These results suggested that miR‐335‐5p overexpression promoted apoptosis, whereas miR‐335‐5p inhibition repressed apoptosis.

## miR‐335‐5p suppresses HNSCC cell metastasis and epithelial–mesenchymal transition (EMT) process

**Fig. 3:**
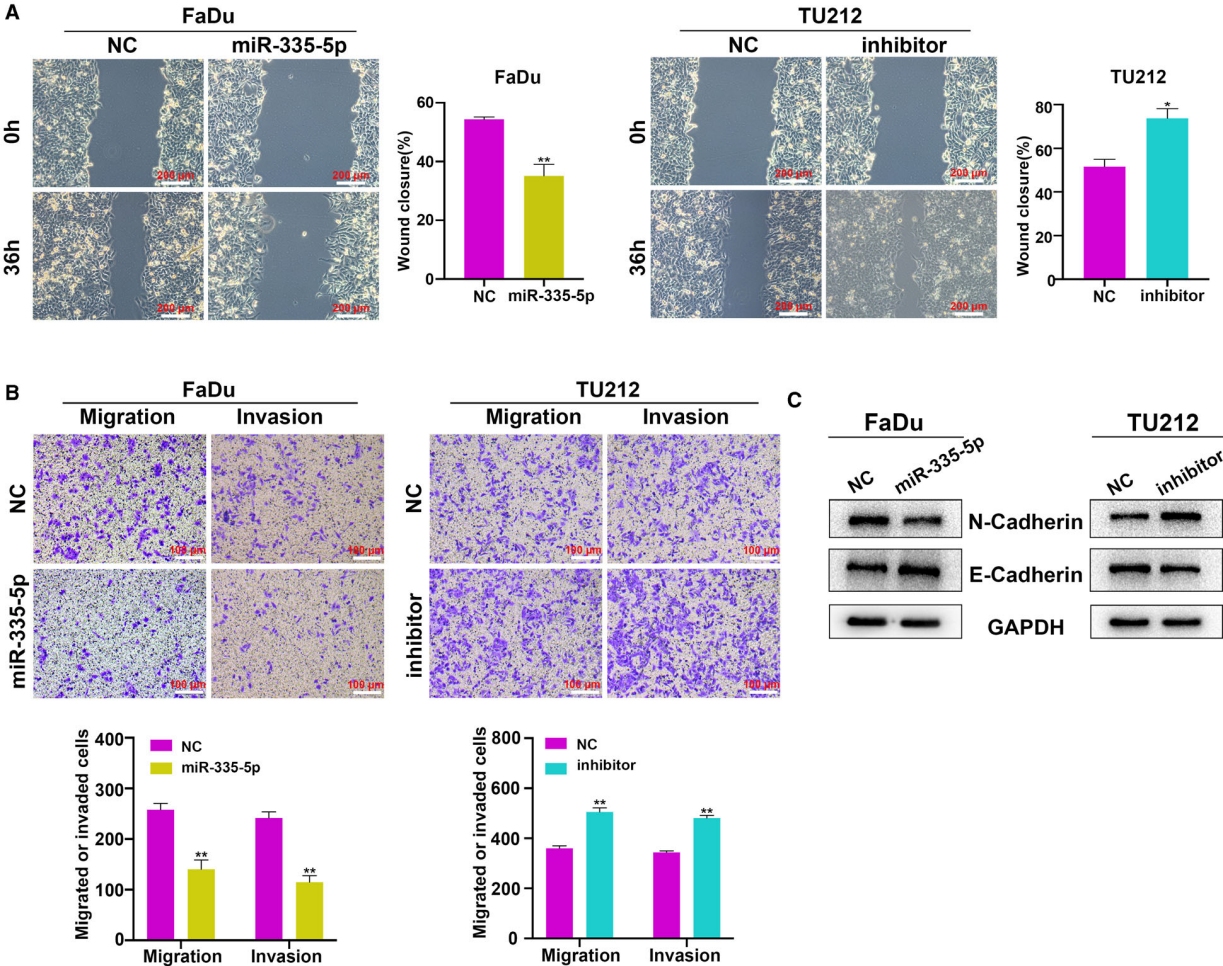
miR‐335‐5p inhibits cell migration, invasion, and EMT process in HNSCC cells.(A, B) The effects of miR‐335‐5p mimic and inhibitor on HNSCC cell migration and invasion as determined by wound‐healing assays (*n = 3*; scale bar: 200 μm) and Transwell assays (*n = 3*; scale bar: 100 μm). (C) N‐Cadherin and E‐cadherin protein expression were detected in HNSCC cells with miR‐335‐5p overexpression and downregulation by western blot assay (*n = 3*). Data are presented as mean ± SD. Comparisons between groups were analyzed using Student's *t*‐test. *P < 0.05, **P < 0.01.

We next conducted wound‐healing and Transwell assays to evaluate the role of miR‐335‐5p in the metastasis of HNSCC cells. Wound‐healing assays showed that miR‐335‐5p overexpression repressed the migration capability of FaDu cells, while increased migration activity was observed in TU212 cells transfected with the miR‐335‐5p inhibitor (Fig. 3A). Additionally, Transwell assays revealed that miR‐335‐5p overexpression dramatically inhibited the migration and invasion of FaDu cells compared with NC, while miR‐335‐5p inhibition promoted migration and invasion in TU212 cells (Fig. 3B). Previous studies showed that EMT contributes to cancer metastasis [20, 21]. miR‐335‐5p overexpression increased the E‐cadherin expression and suppressed N‐cadherin expression in FaDu cells, while the miR‐335‐5p inhibitor induced opposite effects in TU212 cells (Fig. 3C). Collectively, these data demonstrated that miR‐335‐5p inhibits metastasis and EMT of HNSCC cells *in* *vitro*.

## *MAP3K2* is a functional target of miR‐335‐5p

**Fig. 4:**
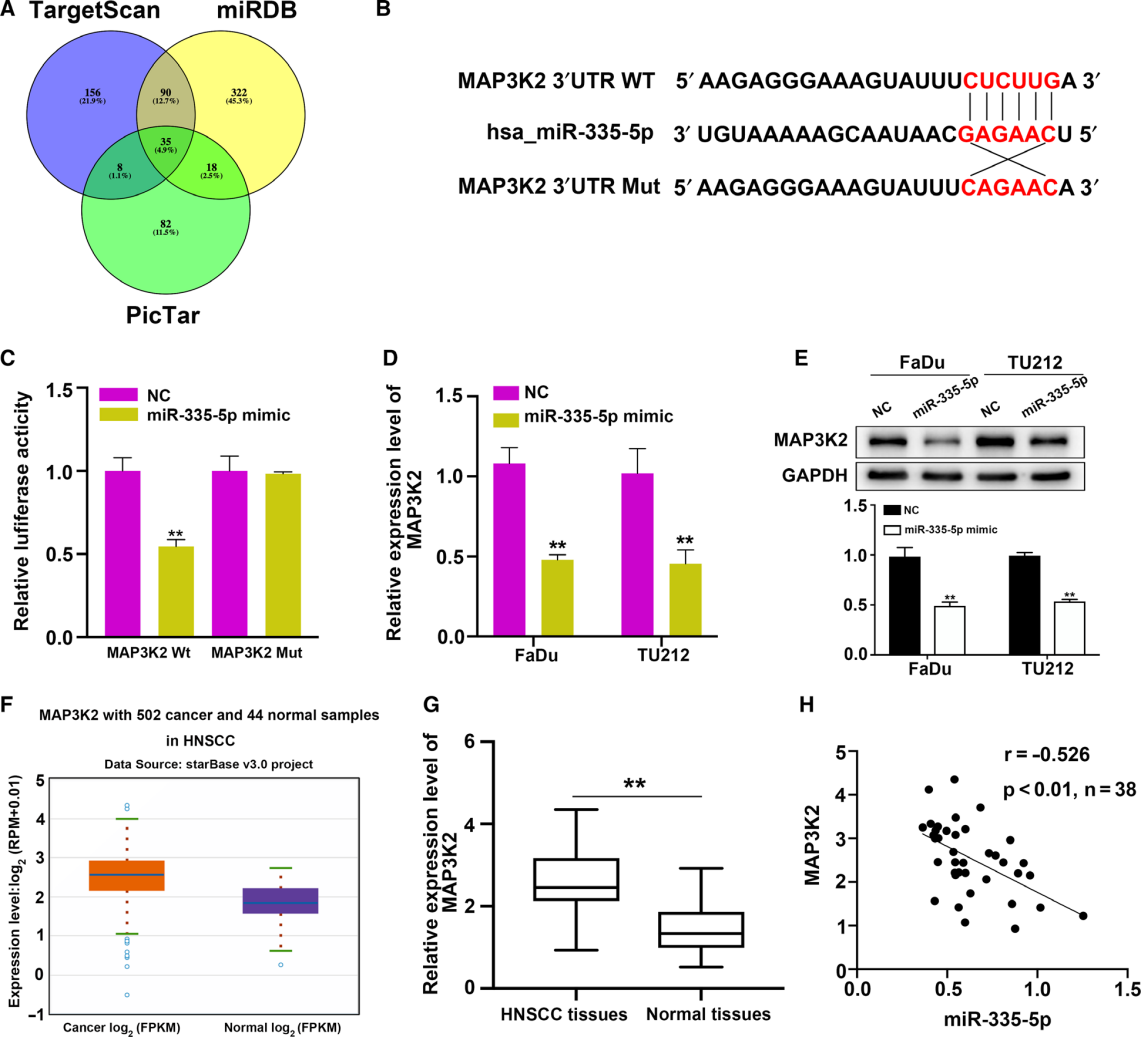
*MAP3K2* is a direct target of miR‐335‐5p in HNSCC. (A) Potential targets of miR‐335‐5p were analyzed by bioinformatic analyses. (B) Predicted miR‐335‐5p target sequences in the 3’UTR of *MAP3K2*. Wild‐type and mutant binding sites for miR‐335‐5p in the 3′UTR of *MAP3K2* in red. (C) Relative luciferase activities in 293T cells transfected with *MAP3K2* 3′UTR wild‐type (WT) or *MAP3K2* 3′UTR‐mutant (Mut) reporter plasmid and miR‐335‐5p mimic or NC (*n = 3*). (D, E) The expression levels of *MAP3K2* mRNA and MAP3K2 protein expression were detected in HNSCC cells after transfection with miR‐335‐5p mimic by qRT–PCR (*n = 3*) and western blot assay (*n = 3*). (F) qRT–PCR was used to examine the expression levels of *MAP3K2* in 502 HNSCC tissues and 44 normal tissues from TCGA and GEO databases. (G) The expression levels of *MAP3K2* were also upregulated in 38 paired of clinical HNSCC tissues and normal tissues. (H) Negative correlation between the expression of miR‐335‐5p and *MAP3K2* in 38 paired of clinical HNSCC tissues. Data are presented as the mean ± SD. Comparisons between groups were analyzed using Student's *t*‐test. **P < 0.01.

To investigate the mechanisms of miR‐335‐5p in HNSCC progression, we searched for putative binding targets of miR‐335‐5p using three bioinformatic softwares, TargetScan (http://www.targetscan.org/), PicTar (https://pictar.mdc‐berlin.de), and miRDB (http://mirdb.org). While more than 400 genes were predicted as potential targets, 35 genes were identified by all three algorithms (Fig. 4A). Our results thus far have indicated an antitumor function for miR‐335‐5p in HNSCC, so we focused on the oncogenes among the 35 potential targets (Table S1). Bioinformatic analyses revealed the binding sites for miR‐335‐5p in the 3′UTR of *MAP3K2* (Fig. 4B). A luciferase reporter assay was then conducted to evaluate the potential regulation of *MAP3K2* by miR‐335‐5p. We found that overexpression of miR‐335‐5p suppressed the luciferase activity of the luciferase vector driven by the wild‐type *MAP3K2* 3′UTR but had no impact on the mutant vector in which the putative binding sites were mutated (Fig. 4C). After transfection with miR‐335‐5p mimic in HNSCC cells, qRT**–**PCR was applied to measure the expression of *MAP3K2*. The results indicated that *MAP3K2* expression was obviously decreased (Fig. 4D). Consistent with the qRT**–**PCR results, miR‐335‐5p overexpression attenuated MAP3K2 protein in HNSCC cells (Fig. 4E). *MAP3K2* expression was increased in HNSCC tissues from the TCGA database and our samples (Fig. 4F,G). Furthermore, we observed a converse relationship between the expression of *MAP3K2* and miR‐335‐5p in 38 pairs of HNSCC tissues (Fig. 4H). Collectively, these results revealed that *MAP3K2* is a functional target of miR‐335‐5p.

## MAP3K2 expression rescues the inhibitory effect of miR‐335‐5p on HNSCC cells

**Fig. 5:**
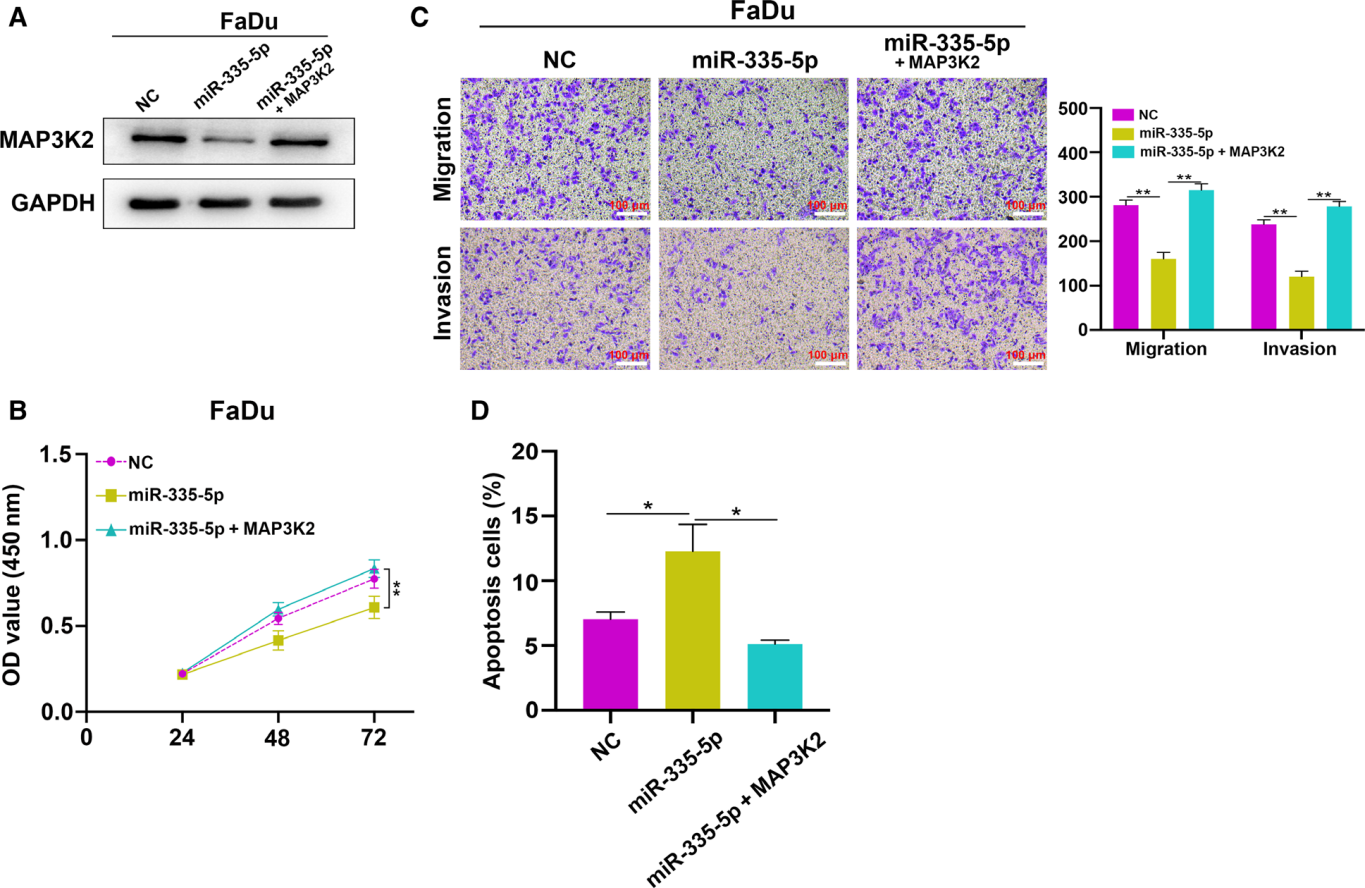
miR‐335‐5p inhibited cell proliferation and metastasis and promoted apoptosis in HNSCC by regulating MAP3K2. (A) Western blot assay indicated that MAP3K2 expression was decreased in cells transfected with miR‐335‐5p mimic and restored in cells cotransfected with pcDNA3.1‐MAP3K2 (*n = 3*). (B, C) CCK‐8 assay (*n = 3*) and Transwell assay (*n = 3*; scale bar: 100 μm) were showed that overexpression of MAP3K2 attenuated miR‐335‐5p inhibition‐mediated effects on cell proliferation, migration, and invasion. (D) Flow cytometry showed that overexpression of MAP3K2 weakened miR‐335‐5p‐mediated effects on HNSCC cell apoptosis (*n = 3*). Data are presented as mean ± SD. Comparisons among groups were analyzed using one‐way ANOVA. *P < 0.05, **P < 0.01.

To evaluate whether MAP3K2 could rescue the effects of miR‐335‐5p on HNSCC cell growth, metastasis, and apoptosis, we performed rescue assays. We confirmed that the miR‐335‐5p‐induced MAP3K2 decrease was recovered upon expression of pcDNA3.1‐MAP3K2 (Fig. 5A). MAP3K2 overexpression partially recovered the suppressive effects of miR‐335‐5p on cell growth (Fig. 5B) and metastasis (Fig. 5C). Furthermore, the increase in apoptotic cells induced by miR‐335‐5p was recovered by coexpression of MAP3K2 (Fig. 5D). These results revealed that miR‐335‐5p might inhibit the progression of HNSCC by attenuating MAP3K2 expression.

## miR‐335‐5p inhibits the HNSCC tumor growth *in* *vivo*

**Fig. 6:**
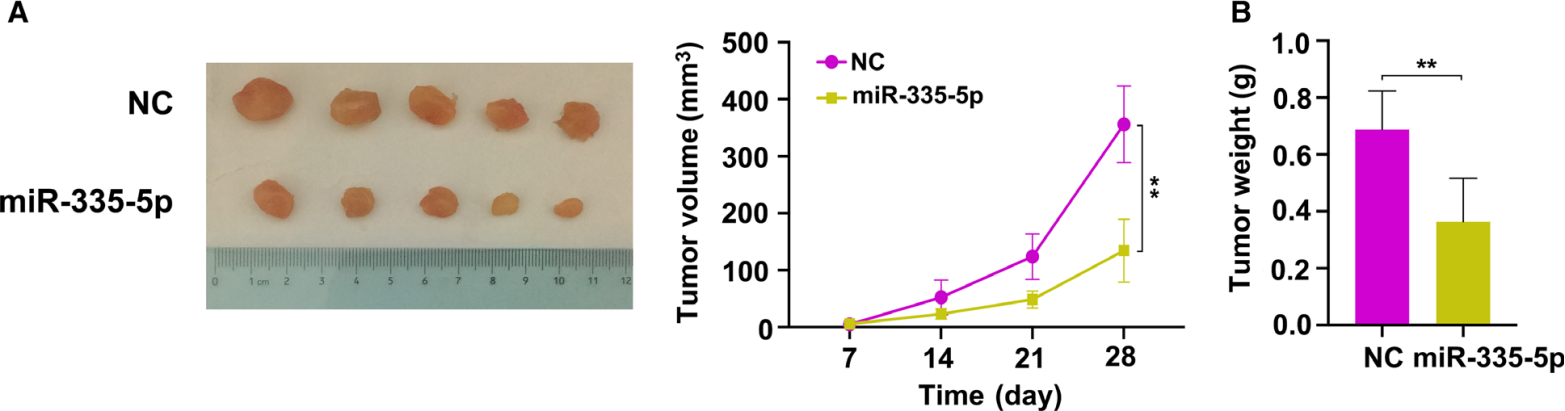
miR‐335‐5p inhibits HNSCC tumor growth *in* *vivo*. (A) The images of xenograft tumors of each group were shown (*n* = 5 in each group); tumor volume was measured every 7 days, and tumor growth curves were plotted. (B) After 1 month, the tumors were dissected and weighed (*n* = 5 in each group). Data are presented as the mean ± SD. Comparisons between groups were analyzed using Student's *t*‐test.   **P < 0.01.

To determine the antitumor function of miR‐335‐5p *in* *vivo*, a subcutaneous tumor xenograft experiment was performed using FaDu cells with miR‐335‐5p overexpression in mice. The tumor volume (Fig. 6A) and weight (Fig. 6B) in the miR‐335‐5p overexpression group were markedly smaller than levels in the control mice. Altogether, these data showed that miR‐335‐5p inhibits HNSCC cell growth *in* *vivo*.

## Discussion

Growing evidence has suggested that aberrant miRNAs may be involved in the tumorigenesis and development of multiple cancers, such as gastric cancer, lung cancer, and HNSCC. MiR‐675 was frequently upregulated, and promoted cell growth and invasion by targeting PITX1 in gastric cancer [22]. MiR‐605‐5p enhanced the proliferation and invasion by targeting TNFAIP3 in non‐small‐cell lung cancer [23]. MiR‐4295 promoted cell growth, migration, and EMT process by targeting NPTX1 in HNSCC [24]. Recent studies showed that miR‐335‐5p was aberrantly expressed in various cancers and showed tumor suppressive functions [14, 15, 16, 17, 18]. For instance, miR‐335‐5p inhibits cell metastasis in gastric cancer [14], and in thyroid cancer, miR‐335‐5p suppresses cell growth and invasion [15]. MiR‐335‐5p is also elevated and shows oncogenic functions in several cancers. For example, miR‐335 expression was increased in colorectal cancer and promoted tumor growth [16]. These studies indicated that the biological function of miR‐335‐5p might depend on the cancer type. However, the biological roles and regulatory mechanisms of miR‐335‐5p in HNSCC remain elusive. Here, we discovered that miR‐335‐5p expression was downregulated in HNSCC tissues in the TCGA and GEO databases and our samples. miR‐335‐5p expression was also inversely associated with advanced TNM stage and lymph node metastasis in HNSCC tissues. Additionally, increased miR‐335‐5p inhibited HNSCC cell growth, migration, and invasion and increased apoptosis *in* *vitro,* while miR‐335‐5p inhibition had the opposite effects. Furthermore, miR‐335‐5p overexpression restrained tumor growth *in* *vivo*. Taken together, these data demonstrated that miR‐335‐5p exhibits tumor suppressor functions in HNSCC and may serve as a potential therapeutic target for HNSCC.

miRNAs regulate multiple biological processes by directly modulating target genes [25]. For instance, miR‐671‐5p suppressed tumor growth by targeting CCND1 and CDC34 in osteosarcoma [26]. MiR‐132‐3p inhibited the migration and invasion via targeting LAPTM4B in breast cancer [27]. MiR‐519c‐3p targeted BTG3 to promote tumor growth and metastasis in hepatocellular carcinoma [28]. Here, we identified *MAP3K2* as a direct target of miR‐335‐5p using three different algorithms. *MAP3K2* is a member of serine/threonine protein kinase family. Previous studies have reported a critical role for *MAP3K2* in tumor development. For instance, miR‐34c‐3p negatively regulates triple‐negative breast cancer invasiveness by modulating the MAP3K2 pathway [29]. Moreover, both miR‐186 and miR‐582‐5p were attenuated and suppressed cell growth and metastasis by regulating MAP3K2 in non‐small‐cell lung cancer [30, 31]. However, the regulatory mechanisms and biological function of *MAP3K2* in HNSCC were unknown. Here, we showed that *MAP3K2* expression was elevated in HNSCC tissues. Increased miR‐335‐5p suppressed MAP3K2 expression in HNSCC cells, and rescue assays showed that MAP3K2 overexpression partly rescued the suppressive effects of miR‐335‐5p on growth, migration, invasion, and apoptosis of HNSCC cells, demonstrating an oncogenic function for *MAP3K2* in HNSCC.

In conclusion, our study demonstrated that miR‐335‐5p expression was reduced in HNSCC and repressed HNSCC cell growth, metastasis, and EMT and promoted cell apoptosis. Furthermore, miR‐335‐5p overexpression suppressed tumor growth *in* *vivo*. Moreover, we identified *MAP3K2* as a novel target of miR‐335‐5p, and MAP3K2 overexpression partially rescued the inhibitory function of miR‐335‐5p. Hence, miR‐335‐5p might function as a therapeutic target for HNSCC patients.

## Conflict of interest

The authors declare no conflict of interest.

## Author contributions

ZXW and LFL designed the project. ZXW, SQY, XMC, and CPH performed all experiments. ZXW, ABZ, and CL analyzed data. ZXW wrote the primary manuscript. LFL reviewed and revised the manuscript. All of the authors read and approved the final manuscript.

## Supporting information


**Table S1**. The potential target genes of miR‐335‐5p.

## Data Availability

DOIs of the public databases in this study are as follows: https://doi.org/10.1093/nar/gkt1248; https://doi.org/10.1093/nar/gkw1079. All data that support the findings of this study are available from the corresponding author upon reasonable request.
