# Supplementary material for: MiR‐335‐5p inhibits the progression of head and neck squamous cell carcinoma by targeting MAP3K2
Source: FEBS Open Bio. 2020 Oct 12;10(11):2282–93. doi: 10.1002/2211-5463.12955 (PMC7609806; doi:10.1002/2211-5463.12955)
Supplement: Supplementary file 1 — Table S1. The potential target genes of miR‐335‐5p. [file FEB4-10-2282-s001.docx]

| **Table S1. The potential target genes of miR-335-5p** | |
| --- | --- |
| **Number** | **Gene Name** |
| 1 | HAND1 |
| 2 | ZMPSTE24 |
| 3 | HOXD8 |
| 4 | RNF141 |
| 5 | CALU |
| 6 | APTX |
| 7 | UBE2G1 |
| 8 | SORCS1 |
| 9 | ARPC5L |
| 10 | PSD3 |
| 11 | RASA1 |
| 12 | RPRM |
| 13 | MAX |
| 14 | SNIP1 |
| 15 | CRIM1 |
| 16 | PPP6C |
| 17 | DAAM1 |
| 18 | NRXN1 |
| 19 | STIM2 |
| 20 | F13A1 |
| 21 | TFDP2 |
| 22 | ADCY3 |
| 23 | NR4A3 |
| 24 | KDELR1 |
| 25 | EEF2K |
| 26 | PPP1R3A |
| 27 | MOSPD1 |
| 28 | PGF |
| 29 | CHFR |
| 30 | CDH11 |
| 31 | SP1 |
| 32 | MLLT3 |
| 33 | MAP3K2 |
| 34 | HPCAL4 |
| 35 | HIC2 |
